# Barakat syndrome diagnosed decades after initial presentation

**DOI:** 10.1530/EDM-23-0018

**Published:** 2023-12-20

**Authors:** Umberto Spennato, Jennifer Siegwart, Britta Hartmann, Elisabeth Julia Fischer, Cecilia Bracco, Joel Capraro, Beat Mueller, Philipp Schuetz, Andreas Werner Jehle, Tristan Struja

**Affiliations:** 1 Medical University Clinic, Division of Endocrinology, Diabetes, and Metabolism, Cantonal Hospital Aarau, Switzerland; 2 Institute for Laboratory Medicine, Division Medical Genetics, Cantonal Hospital Aarau, Switzerland; 3 Medical Faculty of the University of Basel, Switzerland; 4 Department of Internal Medicine, Hirslanden Klinik St. Anna, Lucerne, Switzerland; 5 Transplantation Immunology and Nephrology, University Hospital Basel, Basel, Switzerland

**Keywords:** Adult, Female, White, Switzerland, Kidney, Genetics and mutation, Hypoparathyroidism, New disease or syndrome: presentations/diagnosis/management, December, 2023

## Abstract

**Summary:**

Barakat syndrome, also called HDR syndrome, is a rare genetic disorder encompassing hypoparathyroidism (H), sensorineural deafness (D) and renal disease (R). A 64-year-old woman was referred to our endocrinology clinic for a switch in treatment (from dihydrotachysterol to calcitriol). She had progressive sensorineural deafness since the age of 18 and idiopathic hypoparathyroidism diagnosed at age of 36. Her medical history included osteoporosis with hip/spine fractures, nephrolithiasis and a family history of hearing loss, osteoporosis and kidney disease. The patient’s clinical presentation indicated Barakat syndrome. Genetic analysis found a GATA3:c.916C>T nonsense variant. Further tests such as audiometry, labs and renal imaging supported the diagnosis. Due to rarity and manifold symptoms, diagnosis can be challenging. Optional GATA3 testing was suggested in 2018, except in cases of isolated sensorineural deafness or renal disease with pertinent family history. In isolated ‘H’ cases without ‘D’ and ‘R’, GATA3 studies are not required, as no haploinsufficiency cases were reported. Given the rise in genetic disorders, physicians should consistently consider rare genetic disorders in patients with suggestive symptoms, even decades after onset. Although diagnosis might not always impact management directly, it aids patients in accepting their condition and has broader family implications.

**Learning points:**

- There is currently an important increase in genetic and clinical characterization of new orphan diseases and their causative agents.
- Unbiased re-evaluation for possible genetic disorders is necessary at every consultation.
- It is essential to recognize the differential diagnosis of idiopathic hypoparathyroidism.
- The patient’s clinical presentation and family history can be important to establish the correct diagnosis.
- Physicians should not hesitate to search a patient’s signs and symptoms online.

## Background

Barakat syndrome is a rare, clinically heterogeneous genetic disorder characterized by the triad of hypoparathyroidism (H), sensorineural deafness (D) and renal disease (R) (i.e. HDR syndrome). Barakat syndrome is phenotypically identified with different combinations of the ‘HDR’ triad with or without heterozygous *GATA3* gene variants. The complete ‘HDR’ is found in 64.4% of patients with the syndrome, ‘HD’ in 27.2%, ‘DR’ in 4.4%, ‘R’ in 1.7%, ‘HR’ in 1.7% and ‘D’ in 0.6%, respectively. Due to hypoparathyroidism, patients might present with signs and symptoms of hypocalcaemia, such as seizures, tetany, myalgia and neuromuscular irritability (1). As routine prenatal ultrasound screening and neonatal hearing tests are becoming more common, most patients will be diagnosed presenting with either deafness or renal disease.

HDR syndrome is primarily caused by variants in the *GATA3* gene, located on chromosome 10p (10p14). *GATA3* belongs to a family of dual zinc-finger transcription factors involved in vertebrate embryonic development of the parathyroid glands, auditory system, kidneys, thymus, and central nervous system. Heterozygous *GATA3* variants lead to loss of or reduced DNA-binding affinity or altered DNA binding by conformational changes, causing developmental anomalies (2).

The prevalence of Barakat syndrome is very rare. Up to 2018, 180 patients from different age, ethnic and racial groups have been reported in the literature (1), and since then we identified six additional case reports on PubMed.

## Case presentation

**Figure 1:**
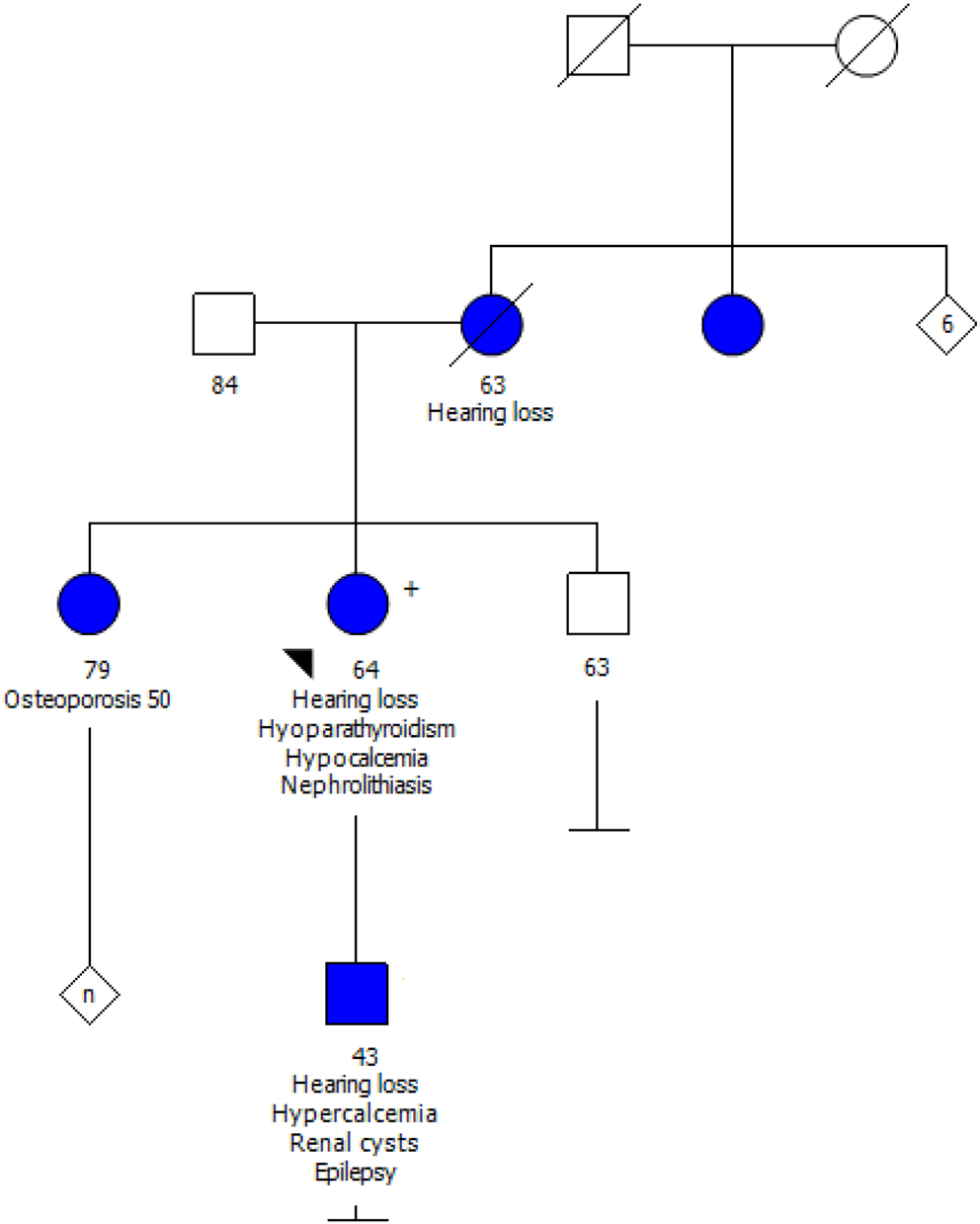
Family pedigree supporting an autosomal dominant inheritance pattern with varying penetrance.

A 64-year-old female with idiopathic hypoparathyroidism was referred to our endocrinology clinic for a switch in treatment from dihydrotachysterol to calcitriol. Hypoparathyroidism manifested when she was 36 years old leading to recurrent nephrolithiasis and intermittent hypercalcaemia due to overtreatment with dihydrotachysterol. Progressive sensorineural deafness started at the age of 18. At the age of 54, she suffered from osteoporotic hip and spine fractures. Over the last decades, she had multiple episodes of recurrent nephrolithiasis requiring surgical intervention. The family history was remarkable for deafness in her mother, and osteoporosis in her sister. She had no dysmorphic features. The patient had a 43-year-old son with progressive hearing loss starting at the age of 11 years and kidney disease (see Fig. 1 for pedigree). Although the son underwent genetic testing, he did not want to share further information regarding his disease course.

## Investigation

Laboratory testing showed elevated plasma calcium and phosphorus due to overtreatment with dihydrotachysterol, low magnesium and low levels in parathormone (see Table 1). As the initial symptoms of our patient manifested almost 30 years ago, previous laboratory values were not retrievable anymore.

**Table 1:** Laboratory values. Blood test results with dihydrotachysterol, vitamin D derivative and calcium carbonate therapy before therapy change. Initial laboratory values were not retrievable anymore as the initial presentation occurred 30 years ago.

Values (unit)	RR	Before	After
Plasma calcium (mmol/L)	2.15–2.55	2.65	2.24
Corrected calcium (mmol/L)	2.15–2.55	2.85	2.37
Magnesium (mmol/L)	0.74–0.99	0.65	0.77
Phosphorus (mmol/L)	0.81–1.62	1.54	1.16
Albumin (g/L)	34–50	32.0	35.0
Parathyroid hormone (ng/L)	18.4–72	6.9	N/A
25-hydroxy vitamin D (nmol/L)	50.0–250	67.3	N/A
Creatinine (μmol/L)	40–90	125	114
eGFR (mL/min/1.73 m^2^)	>60	35	45
Urine calcium (mmol/L)	1.2–5.0	5.6	N/A
Urine creatinine (μmol/L)	7000–18 000	8228	N/A
Urine calcium/creatinine ratio (mg/g)	N/A	241.4	N/A
Urine calcium/creatinine (mmol/mmol)	0.1–0.32	0.285	N/A
Urine pyridinoline/creatinine (nmol/mmol)	40–100	142	N/A
Urine desoxypyridinoline/creatinine (nmol/mmol)	9–20	26.0	N/A

eGFR, estimated glomerular filtration rate; N/A, not available; RR, reference range.

**Figure 2:**
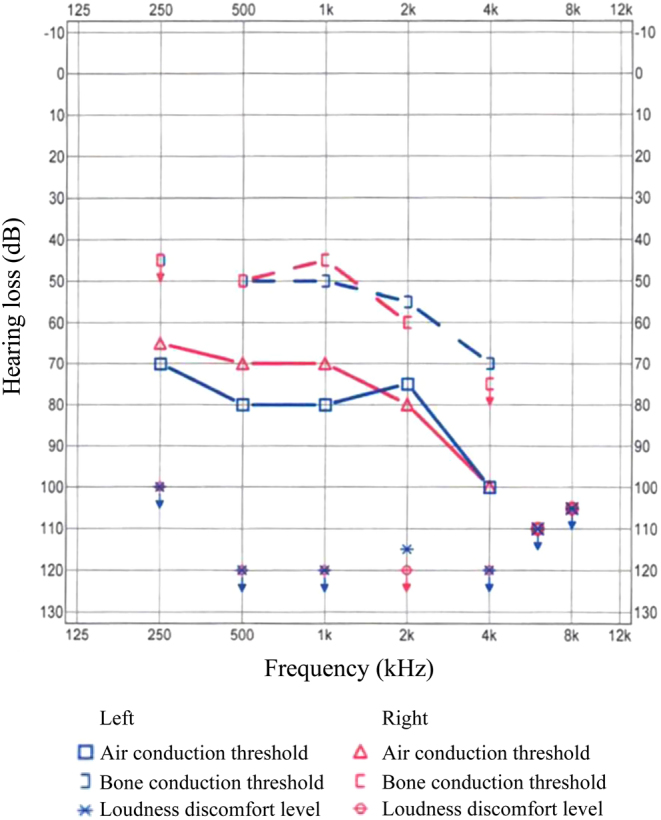
Pure tone audiogram showing air and bone conduction threshold for each ear in decibels (dB) of hearing loss (normal hearing at 0 dB) and frequency in kilohertz (kHz). There is a severe hearing loss of about 80 dB on the right and 85 dB on the left more pronounced at higher frequencies.

Ultrasonography of the kidneys demonstrated no morphological defect. Echocardiography and electrocardiography findings were normal. Pure tone audiogram testing obtained earlier revealed severe sensorineural deafness, with hearing losses of 80 dB (right ear) to 85 dB (left ear) more pronounced at higher frequencies on both ears (see Fig. 2) with consequent bilateral implantation of Bone Anchored Hearing Aid (BAHA) devices.

Based on laboratory findings, clinical manifestation, and personal history, we performed an online search and confirmed the diagnosis of HDR syndrome. Informed consent for genetic analysis was obtained.

The coding regions (exons 3–6) and adjacent intronic regions of the *GATA3* gene (transcript number NM_001002295.2) were analysed using Sanger sequencing. Due to technical limitations, exon 2 of the *GATA3* gene was not covered. Sequences were mapped to the reference genome and gene variants were assessed if their allelic frequency was less than 1% in the general population according to the GnomAD database. Variants in the 5′- and 3′-untranslated region (UTR) and solely intronic variants outside of splice sites were not assessed. We excluded larger duplications or deletions in the *GATA3* gene using multiplex ligation-dependent probe amplification (Salsa MLPA kit P234-A3, MRC-Holland).

**Figure 3:**
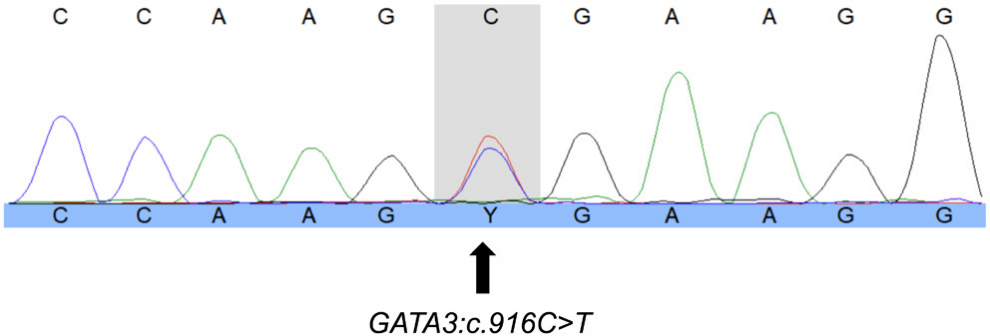
Direct sequencing identifies the heterozygous variant, substitution of cytosine for thymine in complementary DNA (cDNA).

**Figure 4:**
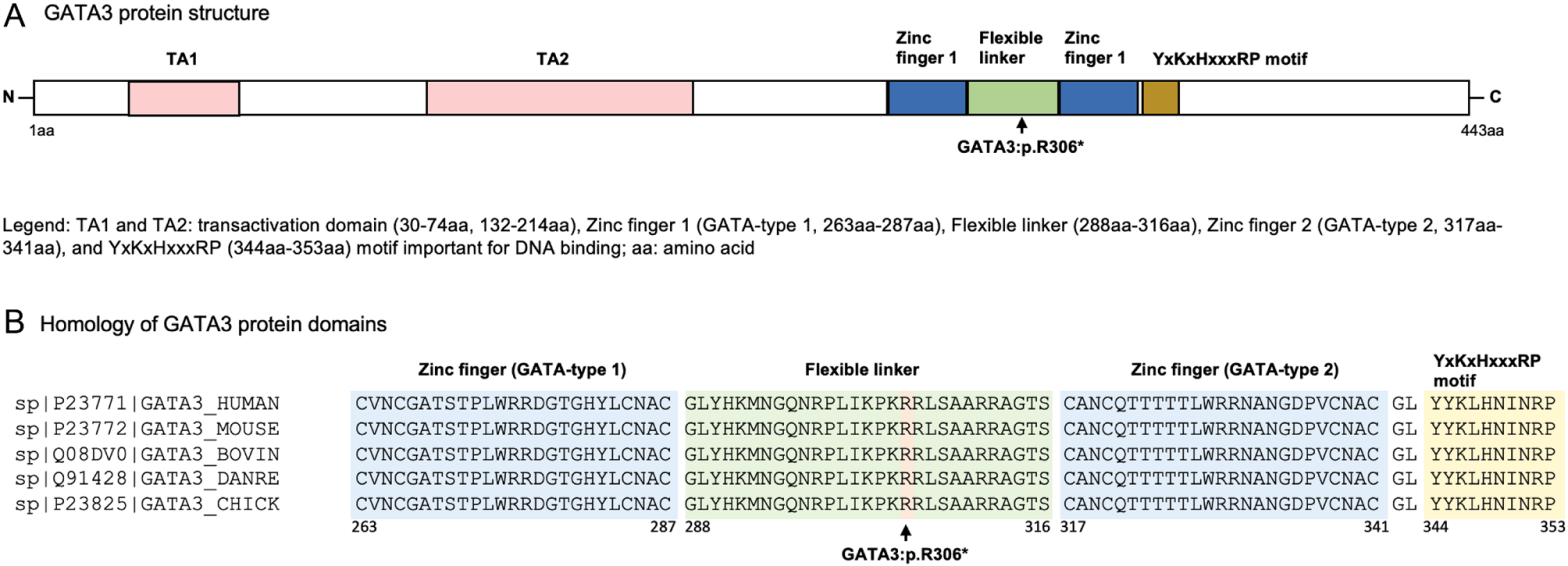
(A) GATA 3 protein structure showing the location of the reported mutation. (B) Protein homology of GATA 3 between *Homo sapiens* (human), *Mus musculus* (mouse), *Bos taurus* (bovine), *Danio rerio* (danre), and *Gallus gallus* (chick) and the location to the reported mutation.

Above analysis showed that the patient carries a heterozygous pathogenic variant (see Fig. 3). *In* *silico* analysis on UniProt showed that the *GATA3* protein structure in which this mutation occurred is highly conserved between various species being an indication of its pathogenicity (see Fig. 4) (1, 2).

## Outcome and follow-up

After diagnosing the patient with Barakat syndrome, she visited our clinic regularly for calcium and phosphate measurements.

## Discussion

The clinical manifestation of deafness, hypoparathyroidism, and kidney disease is a very rare combination and raised suspicions of a common underlying disease process. When further inquiring on family history, our patient told us about similar signs and symptoms in her mother and son. This led us to perform a literature search on potential genetic disorder that could explain the clinical presentation.

### Genetic background

Our case of Barakat syndrome is an excellent example of the phenotypic variability of heterozygous *GATA3* variants.

The identified heterozygous variant *GATA3* c.916C>T in exon 4 is a scarce nonsense variant (not found in the population database GnomAD) and results in a premature stop codon (p.(Arg306*)) located in the flexible linker between the two zinc finger domains of the protein.

Thus, the variant might lead to a truncated protein lacking the C-terminal zinc finger domain, or loss or reduced DNA binding affinity by conformational changes causing developmental anomalies. Similar nonsense variants have already been reported as pathogenic (3).

When writing the initial report, this variant was neither listed in the databases ClinVar, HGMD, nor in the scientific literature. Earlier this year, the variant was entered with a single submission as pathogenic in the ClinVar database; however, no functional data are available. According to the ACMG/ACGS criteria, this variant should be classified as likely pathogenic (class 4) (3).

Similar to our study, Van Esch *et al.* identified a nonsense variant resulting in a stop codon in exon 4 of *GATA3* (c.828C>T, p.(Arg277*), heterozygote) in patients with hypoparathyroidism and bilateral sensorineural deafness but without renal anomalies, suggesting a reduced penetrance for renal anomalies of nonsense variants (4).

Ali *et al.* grouped *GATA3* variants into three classes based upon their functional consequences on DNA binding (2). The first class leads to, particularly truncated variants resulting in loss of DNA binding due to loss of the carboxyl-terminal zinc finger and accounts for more than 90% of all variants. The second class reduces DNA binding affinity, and the third class does not alter DNA binding or affinity but is probably associated with conformational alterations (2). Our nonsense variant should be considered part of the first class; however, functional studies would be needed to confirm this.

In general, genetic syndromes raise the question whether treatment should be different from similar, more common non-genetic manifestations.

### Primary hypoparathyroidisms and calcium disorders

HDR syndrome alongside other genetic diseases comprises 10% of all cases of hypoparathyroidism. Under physiological conditions, hypocalcaemia leads to a reduction in occupancy of calcium-sensing receptors triggering an increase in the synthesis and secretion of parathormone (5).

On the other hand, hypocalcaemia causes neuromuscular irritability, seizures, tetany or paraesthesia (5). Our patient experienced paraesthesia, abdominal pain accompanied by vomiting and diarrhoea at serum calcium levels below 1.5 mmol/L (reference range, 2.15–2.5 mmol/L).

Management of hypoparathyroidism is based on substitution with calcium and an active form of vitamin D. Another possibility is the use of recombinant human parathormone rh-PTH 1-84 once daily (5).

Besides symptomatic hypocalcaemia, prolonged insufficient treatment of hypoparathyroidism may lead to structural and morphological skeletal changes (6). In a review of the literature, we did not find evidence for an increased risk for cardiovascular disease and malignancy, but possibly a slightly increased risk for upper extremity fractures (7). Our patient has a history of osteoporosis with numerous fractures (e.g. ribs, radius, spine). Antiresorptive therapy with denosumab was established for 10 years, and we recommended a change to a bisphosphonate for an additional year to mitigate the risk of rebound bone loss after stopping denosumab. We are not aware of any studies investigating osteoporosis under treatment with parathormone, for example teriparatide or abaloparatide in hypoparathyroidism, but given the clear therapeutic benefit in osteoporosis, its use in hypoparathyroidism is even more appealing (5).

In our patient, the initial serum calcium concentration was above the reference range (i.e. ≥2.8 mmol/L), due to the overly aggressive substitution with dihydrotachysterol and calcium carbonate. After switching to calcitriol 0.25 mg twice daily and adding 12.5 mg hydrochlorothiazide once daily – to increase serum calcium, decrease hypercalciuria and subsequent nephrocalcinosis – calcium levels stabilized in a tolerable range (see Table 1) (5).

Mitchell *et al.* corroborated the relationship between hypoparathyroidism and kidney disease in a cross-sectional study of more than 100 patients with hypoparathyroidism. They showed that 40% of patients had chronic renal disease and up to one-third had evidence of renal calcification in ultrasound and/or computed tomography imaging (8).

### Kidney

The renal penetrance of HDR syndrome is variable. Reports indicate that up to 41% of patients have either aplasia, hypoplasia, or dysplasia; 16% have vesicoureteral reflux; and 11% have cysts or pelvicalyceal deformities (9). Although our patient had CKD stage 3 renal insufficiency, there were no pathological findings in imaging studies and kidney function was stable over time. We did not measure the albumin–creatinine ratio in urine. However, Barakat syndrome can present itself as a steroid-resistant nephrotic syndrome which was already recognized in the initial case description (1).

*GATA3* is strongly expressed in two major progenitor populations, ureteric bud and stroma cells, and plays a crucial role in kidney development which explains the occurrence of renal dysplasia. Additionally, human fetal and adult kidneys demonstrated a strong *GATA3* expression in mesangial progenitor cells as well as in mature mesangial cells, respectively (10). Heterozygous *GATA3* knockout mice showed reduced proliferation rates of mesangial cells possibly leading to defective repair processes and *GATA3* was shown to be increased in rodent models of mesangioproliferative glomerulonephritis and in patients with IgA nephropathy (9, 10). These observations may indicate that GATA3 plays a critical role to maintain glomerular homeostasis.

Hypomagnesaemia can be a subtle part of syndrome’s renal presentation. In our case, hypomagnaesemia was not a predominant finding, since we did not measure the fractional excretion of magnesium. However, Alkaissi *et al.* demonstrated that patients with HDR syndrome can have fractional excretion of magnesaemia up to 9% compared to 2–4% as in the normal population (11).

As the prognosis of affected patients usually depends on the severity of renal disease, early diagnosis and close follow-up are crucial in improving the prognosis.

### Deafness

While renal penetrance is varying, penetrance of sensorineural deafness seems to be high, progressively increasing over the years, and more pronounced at higher frequencies.

A study reported detailed auditory and vestibular phenotypes of six patients with *GATA3* mutations (12). While all patients present with early-onset sensorineural hearing loss and absent distortion product otoacoustic emissions (DPOAEs) in both ears, their auditory brainstem responses were normal, supporting a cochlear site of lesion with a lack of evidence for a vestibular dysfunction.

*GATA3* is involved in the early morphogenesis of the pro-sensory domain of the inner ear and regulates the apoptosis of spiral ganglion neurons during maturation of the auditory system. A knockout in *GATA3* leads to a limited genesis of hair cells and therefore causes severe inner ear defects (12).

## Conclusion

Currently, orphan diseases are increasingly recognised. Physicians should re-evaluate patients with suggestive symptoms for the possibility of rare genetic disorders at every consultation, even decades after their initial manifestation. Although a diagnosis may not always directly influence management, it may help patients to come to terms with their situation and can have consequences for the wider family.

## Declaration of interest

There is no conflict of interest that could be perceived as prejudicing the impartiality of the case study reported.

## Funding

TS was supported by an unrestricted educational grant of the Swiss National Science Foundationhttp://dx.doi.org/10.13039/100000001 (P400PM_194497/1).

## Patient consent

Written informed consent was obtained from the patients for the publication of all personal information contained in this case report.

## Author contribution statement

US, TS and AWJ designed the study. US and TS wrote the first draft of the manuscript and were responsible for the decision to submit the manuscript. All authors provided comments on drafts and approved the final version of the manuscript.

## References

[1] Barakat AJ Raygada M & Rennert OM. Barakat syndrome revisited. American Journal of Medical Genetics 2018 176 1341–1348. (10.1002/ajmg.a.38693) 29663634

[2] Ali A Christie PT Grigorieva IV Harding B Van Esch H Ahmed SF Bitner-Glindzicz M Blind E Bloch C Christin P, Functional characterization of GATA3 mutations causing the hypoparathyroidism-deafness-renal (HDR) dysplasia syndrome: insight into mechanisms of DNA binding by the GATA3 transcription factor. Human Molecular Genetics 2007 16 265–275. (10.1093/hmg/ddl454) 17210674

[3] Richards S Aziz N Bale S Bick D Das S Gastier-Foster J Grody WW Hegde M Lyon E Spector E, Standards and guidelines for the interpretation of sequence variants: a joint consensus recommendation of the American College of Medical Genetics and Genomics and the Association for Molecular Pathology. Genetics in Medicine 2015 17 405–424. (10.1038/gim.2015.30) 25741868 PMC4544753

[4] Van Esch H Groenen P Nesbit MA Schuffenhauer S Lichtner P Vanderlinden G Harding B Beetz R Bilous RW Holdaway I, GATA3 haplo-insufficiency causes human HDR syndrome. Nature 2000 406 419–422. (10.1038/35019088) 10935639

[5] Bilezikian JP. Hypoparathyroidism. Journal of Clinical Endocrinology and Metabolism 2020 105 1722–1736. (10.1210/clinem/dgaa113) 32322899 PMC7176479

[6] Rubin MR Dempster DW Zhou H Shane E Nickolas T Sliney J Jr Silverberg SJ & Bilezikian JP. Dynamic and structural properties of the skeleton in hypoparathyroidism. Journal of Bone and Mineral Research 2008 23 2018–2024. (10.1359/jbmr.080803) 18684087 PMC2686925

[7] Clarke BL Brown EM Collins MT Jüppner H Lakatos P Levine MA Mannstadt MM Bilezikian JP Romanischen AF & Thakker RV. Epidemiology and diagnosis of hypoparathyroidism. Journal of Clinical Endocrinology and Metabolism 2016 101 2284–2299. (10.1210/jc.2015-3908) 26943720 PMC5393595

[8] Mitchell DM Regan S Cooley MR Lauter KB Vrla MC Becker CB Burnett-Bowie SA & Mannstadt M. Long-term follow-up of patients with hypoparathyroidism. Journal of Clinical Endocrinology and Metabolism 2012 97 4507–4514. (10.1210/jc.2012-1808) 23043192 PMC3513540

[9] Belge H Dahan K Cambier JF Benoit V Morelle J Bloch J Vanhille P Pirson Y & Demoulin N. Clinical and mutational spectrum of hypoparathyroidism, deafness and renal dysplasia syndrome. Nephrology, Dialysis, Transplantation 2017 32 830–837. (10.1093/ndt/gfw271) 27387476

[10] Grigorieva IV Oszwald A Grigorieva EF Schachner H Neudert B Ostendorf T Floege J Lindenmeyer MT Cohen CD Panzer U, A novel role for GATA3 in mesangial cells in glomerular development and injury. Journal of the American Society of Nephrology 2019 30 1641–1658. (10.1681/ASN.2018111143) 31405951 PMC6727249

[11] Alkaissi HR & Banerji MA. Primary hypoparathyroidism presenting as idiopathic intracranial hypertension in a patient with Barakat syndrome. Cureus 2022 14 e24521. (10.7759/cureus.24521) 35651450 PMC9138397

[12] Kita M Kuwata Y & Usui T. Familial congenital choanal atresia with GATA3 associated hypoparathyroidism-deafness-renal dysplasia syndrome unidentified on auditory brainstem response. Auris, Nasus, Larynx 2019 46 808–812. (10.1016/j.anl.2018.10.005) 30396722

